# Quality of life and survival outcome for patients with nasopharyngeal carcinoma treated by volumetric-modulated arc therapy versus intensity-modulated radiotherapy

**DOI:** 10.1186/s13014-020-01532-4

**Published:** 2020-04-19

**Authors:** Tai-Lin Huang, Ming-Hsien Tsai, Hui-Ching Chuang, Chih-Yen Chien, Yu-Tsai Lin, Wen-Ling Tsai, Fu-Min Fang

**Affiliations:** 1 Department of Hematology and Oncology, Kaohsiung Chang Gung Memorial Hospital and Chang Gung University College of Medicine, Kaohsiung, Taiwan; 2 Department of Otolaryngology, Kaohsiung Chang Gung Memorial Hospital and Chang Gung University College of Medicine, Kaohsiung, Taiwan; 3 Department of Cosmetics and Fashion Styling, Center for Environmental Toxin and Emerging-Contaminant Research, Cheng Shiu University, Kaohsiung, 83347 Taiwan; 4 Department of Radiation Oncology, Kaohsiung Chang Gung Memorial Hospital and Chang Gung University College of Medicine, No. 123 Ta-Pei Rd., Niao Sung District, Kaohsiung, Taiwan

**Keywords:** Nasopharyngeal carcinoma, Quality of life, Volumetric-modulated arc therapy, Intensity-modulated radiotherapy, EORTC

## Abstract

**Background:**

To evaluate the longitudinal changes of quality of life (QoL) and survival in patients with nasopharyngeal carcinoma (NPC) treated by volumetric-modulated arc therapy (VMAT) versus intensity-modulated radiotherapy (IMRT).

**Methods:**

One hundred and forty non-distant metastatic NPC patients treated by VMAT (*n* = 66) or IMRT (*n* = 74) with simultaneously integrated boost between March 2013 and December 2015 at a single institute were analyzed. QoL was prospectively assessed by the EORTC QLQ-C30 and HN35 questionnaires at the four time points: before RT, RT 42.4 Gy (20 fractions), and 3, 12 months after RT.

**Results:**

The 3-year locoregional relapse-free survival, distant metastasis-free survival, failure-free survival, and overall survival rates were 96.6, 89.4, 86.1%, and 87.4 for the VMAT group, respectively, compared with 91.4, 90.0, 79.8, and 91.3% for the IMRT group (*p* value > 0.05). The pattern of QoL changes was similar between the VMAT and IMRT group. No statistically or clinically significant difference in all the QoL scales was observed between VMAT and IMRT group at each time point. Compared to before RT, we observed statistically (*p*<0.05) and clinically (difference of mean scores≧10) better outcome in global QoL and social functioning, but worse head and neck symptomatic outcome in swallowing, taste/smell, opening mouth, dry mouth, and sticky saliva at the time point of 1 year after RT for both groups.

**Conclusion:**

The study provides the evidence that the tumor control, survival and changes of QoL is compatible for NPC patients treated by VMAT versus IMRT.

## Background

Over the past decades, advances in modern radiotherapy (RT) techniques for treating nasopharyngeal carcinoma (NPC) have emerged with the development of intensity-modulated radiotherapy (IMRT) or, more recently, volumetric-modulated arc therapy (VMAT). As opposed to the fixed gantry beams used in classical IMRT, VMAT is a novel radiation treatment technique based on volumetric modulated rotational delivery. Dual arc VMAT has been reported to be technically superior, e.g. faster delivery time, use of fewer monitor units (MUs) compared to seven-field fixed beam IMRT in NPC patients, and a dosimetric advantage regarding target volume coverage or sparing some organs at risk (OARs) [1–3].

Strong evidence has shown that the dosimetric improvement conferred by the technical advance from conventional 2D RT to IMRT in NPC could transfer to clinical benefit, not only regarding toxicity and tumor control, but also in terms of the patient’s QoL and survival [4–6]. However, whether the therapeutic window of improved QoL or survival in NPC patients could be widened by further technical evolutions of modern RT techniques needs to be evaluated. As far as we know, a clinical comparison has not been reported regarding tumor control, patient survival, or QoL between VMAT and IMRT in NPC patients. In current study, longitudinal results of QoL from NPC patients treated by VMAT or IMRT at a single institution were presented. The data pertaining to QoL were longitudinally collected using the questionnaires of the European Organization for Research and Treatment of Cancer (EORTC) QLQ-C30 and HN35 module at the four time points: before RT (ti1), during RT (42.4 Gy, 20 fractions, ti2), and 3 months (ti3), and 12 months after RT (ti4). We compared the locoregional relapse-free survival (LRRFS), distant metastasis-free survival (DMFS), failure-free survival (FFS) and overall survival (OS), as well as the changes of the EORTC QoL scales at the four time points for NPC patients treated by VMAT versus IMRT.

## Materials and methods

### Patient

After implementation of IMRT system in March 2002, seven-field fixed beam IMRT has become the standard technique in radically treating NPC at the institute. In March 2013, the dual arc VMAT was introduced to treat NPC after installation of the planning system. Patients were eligible for this study if they had a new diagnosis of non-distant metastasis NPC and were curatively treated by IMRT or VMAT with simultaneous integral boost planning for the whole course between March 2013 and December 2015. Other inclusion criteria were age < 80 years, completion of the prescribed RT course, no previous or synchronous malignancies, no cognitive impairment and agreement to complete the Taiwan Chinese version of the EORTC QoL questionnaires. During the study period, 140 patients met the inclusion criteria and informed consent was obtained. The choice of IMRT (*n* = 74) or VMAT (*n* = 66) was nonrandomized and the decision to use IMRT or VMAT techniques was individualized on the basis of physician discretion and mostly based on the availability of the Linac machines.

### RT technique

The technical details of IMRT and VMAT with simultaneous integral boost planning for NPC in the institute have been reported previously [7, 8]. The immobilization, target definition and delineation, and the prescription of dose/fractionation were identical in both techniques. All patients underwent computed tomography (CT)-planned simulation and received the continuous treatment course with one fraction per day and 5 fractions per week. Computerized optimization was used with the fusion of MRI and/or PET with treatment planning CT images, when possible, to accurately delineate the gross tumor volume (GTV), which included the primary disease and nodes greater than 1 cm in diameter or nodes with necrotic centers. Three different dose levels of clinical target volumes (CTVs) were created. The high dose level of CTV (CTV-H) was defined as the GTV with an isotropic extension of 5 mm. The middle dose level of CTV (CTV-M) covered the CTV-H plus the areas at risk for microscopic involvement, including the entire nasopharynx, parapharyngeal space, skull base, retropharyngeal lymph nodes, and bilateral upper neck nodes. The low dose level of CTV (CTV-L) included the CTV-M plus bilateral lower neck nodes. To overcome organ motion and daily treatment set-up uncertainties, the orthogonal KV X-ray images were routinely used to perform the daily image guidance and a planning target volume (PTV) was added with additional margins of 3 to 5 mm on each of the CTVs. The prescribed dose and fractionation for PTV-H, PTV-M, and PTV-L were 69.96 Gy, 59.40 Gy, and 52.80 Gy in 33 fractions, respectively. The delineations of the OARs and constrains of the dosage applied to OARs were under the framework of Radiation Therapy Oncology Group (RTOG) 0225 protocol [9].

The Philips Pinnacle Planning System version 9.2 (Philips, Fitchburg, WI) was used for treatment planning. For VMAT planning, it consisted of dual coplanar arcs of 360° and was simultaneously optimized to be delivered with opposite rotation (clockwise and counter clockwise). There were a total of 182 control points, with the collimator angle of 10–15°. Continuous gantry motion, dose-rate variation and the motion of multi-leaf collimators (MLC) were approximated by optimizing individual beams at 2–4° gantry angle increments. The collapsed cone convolution algorithm was used for dose calculations and SmartArc module was adopted for dose optimization. For IMRT planning, it was designed according to the step-and-shoot methods basically with seven fixed coplanar gantry beams in most cases. Eleven patients required one or two more beams from non-coplanar directions because the tumor was located nearby the brain stem or eyeballs, which necessitated better dose coverage. A collapsed-cone convolution algorithm was used for dose calculations, with a dose grid resolution of 4 mm. The minimum segment area was set to 5 cm^2^, and minimum segment MU was 5 MUs. Direct machine parameter optimization module was adopted for plan optimization. For both planning techniques, the beam delivery was generated with 6-MV photons by the Linac machines equipped with dynamic MLC. All treatment plans were evaluated to ensure that 95% of all the PTVs received the prescription dose. The dose volume histograms of PTVs and OARs were quantitatively assessed and the isodose curves on axial CT slices were qualitatively inspected for each IMRT or VMAT plan.

### Chemotherapy

Patients with stage II to IVB received concurrent chemotherapy with weekly cisplatin 30–40 mg/m^2^ administered during RT courses. Adjuvant chemotherapy with cisplatin 70–80 mg/m^2^ on day 1 and 5-fluorouracil 700–800 mg/m^2^/d on days 1–4 were administered every 3–4 weeks was given for 1–4 cycles to those patients with residual tumor or persistent elevation of plasma EBV-DNA titer.

### QoL assessment

QoL was assessed by the Taiwan Chinese versions of EORTC QLQ-C30 and HN35 questionnaires. The questionnaires have been tested in Taiwanese NPC patients, and excellent reliability and validity were obtained [10]. The EORTC QLQ-C30 is a widely used questionnaire. It incorporates a range of QoL issues relevant to a broad range of cancer patients. It has been translated into many languages and validated for many types of cancer, including head and neck cancer (HNC). It contains a global QoL scale, five functional scales (physical, role, cognitive, emotional, and social), three symptom scales (fatigue, pain, and nausea/vomiting), and six single items (dyspnea, insomnia, appetite loss, constipation, diarrhea, and financial difficulties). The HN35 is a module used for assessing the QoL for head and neck cancer (HNC) patients. It incorporates seven multiple-item scales that assess the symptoms of pain in the head and neck, swallowing ability, senses (taste/smell), speech, social eating, social contact, and sexuality. Also included are six single-item scales, which survey the presence of symptomatic problems associated with teeth, mouth opening, dry mouth, sticky saliva, coughing, and feeling ill. All scales pertaining to the EORTC QLQ-C30 and HN35 range from zero to 100. A high score for a functional or global QoL scale represents a relatively high/healthy level of functioning or global QoL, whereas a high score for a symptom scale represents the presence of a symptom or problem(s) [11, 12].

### Follow-up

Patients were regularly followed up after RT until death or their last follow-up appointment. They were scheduled to visit the clinics at 3 months, and 4- to 6-month intervals in the first two, and third to fifth years, respectively. The median followed-up months were 38 months (range, 12 to 58 months) in the VMAT group, and 46 months (range, 2 to 59 months) in the IMRT group, respectively. Physical and nasopharyngoscopic examinations were routinely performed on every visit. Head and neck MRI scans were performed periodically in the first 5 years or when there were clinical indications. Locoregional failure was determined based on pathologic diagnosis or progressive deterioration shown on consecutive image studies.

### Statistical analysis

The primary endpoint was to compare the mean scores of the QoL scales at each time point between the two study groups and further investigate the results of LRRFS, DMFS, FFS, and OS between them. The duration of survival was calculated from the last day of RT. Patients alive on the last day of follow-up were censored. Survival curves were estimated by the Kaplan-Meier method and log rank test used to compare the statistical difference between survival curves. The Cox proportional hazards regression model (backward) was used for multivariate analysis. The mean scores of the QoL scales were calculated according to the EORTC QLQ scoring manual [13]. To deal with the missing data, the missing items were assumed to have values equal to the average of those items that were present for the respondent, if at least half of the items from the scale have been answered (i.e., mean imputation). A t test was used to compare the mean scores between the two groups at each time point, with a *p* value < 0.05 from the two sided test regarded to be statistically significant. According to the advice from Osoba et al., a 10-point difference on a scale of 0–100 was regarded to be clinically significant [14]. Multivariate ANOVA for repeated measures was used for the comparisons of the changes of QoL scales over the four time points, and the influence of medical- or socio-demographic- variables on these changes were studied by entering them as between-subject factors in the multivariate ANOVA procedure. The software, Microsoft SPSS version 22, was used for data processing.

## Results

### Patient characteristics

The socio-demographic-, tumor-, and treatment- related characteristics of patients are presented in Table 1. No imbalances were found between the two groups in any covariate. The median (range) value of GTV was 51 ml (2–285 ml). Approximately 92% of the patients were stage II-IVb. The completion rate of the QoL assessment was 100, 97.8, 92.8, and 74.3% at t1 to t4, respectively. To study whether patients who dropped out because of noncompliance or death at each time point introduced selection bias, we compared the medical data for those with and without dropouts respectively at each time point between the two groups, and no statistically significant difference was observed.

**Table 1:** Patient characteristics

	All N (%)	IMRT N (%)	VMAT N (%)	*P*
Age, median (range), year	51 (15–78)	51 (15–72)	52 (19–78)	0.26
≦40	29 (20.7)	19 (25.7)	10 (15.2)	
41–65	99 (70.7)	50 (67.5)	49 (74.2)	
> 65	12 (8.6)	5 (6.8)	7 (10.6)	
Gender				0.46
Male	100 (71.4)	55 (74.3)	45 (68.2)	
emale	40 (28.6)	19 (25.7)	21 (31.8)	
Marital status				0.85
Without spouse	42 (30.0)	23 (31.1)	19 (28.8)	
With spouse	98 (70.0)	51 (68.9)	47 (71.2)	
Education years				0.24
≦6	31 (22.1)	17 (23.0)	14 (21.2)	
6 ~ 12	73 (52.1)	34 (45.9)	39 (59.1)	
> 12	36 (25.8)	23 (31.1)	13 (19.7)	
Charlson comorbidity index				0.73
0	78 (55.7)	40 (54.1)	38 (57.6)	
≧1	62 (44.3)	34 (45.9)	28 (42.4)	
Plasma EBV-DNA				1.00
<1500 copies/ml	119 (85.0)	63 (85.1)	56 (84.8)	
≧1500 copies/ml	21 (15.0)	11 (14.9)	10 (15.2)	
Gross tumor volume, ml				0.50
<51 (median)	74 (52.9)	37 (50.0)	37 (56.1)	
≧51 (median)	66 (47.1)	37 (50.0)	29 (43.9)	
WHO histology				0.61
Type IIA	76 (54.3)	42 (56.8)	34 (51.5)	
Type IIB	64 (45.7)	32 (43.2)	32 (48.5)	
AJCC stage				0.43
I	11 (7.9)	8 (10.8)	3 (4.5)	
II	37 (26.4)	20 (27.1)	17 (25.8)	
III	40 (28.6)	22 (29.7)	18 (27.3)	
IVA-B	52 (37.1)	24 (32.4)	28 (42.4)	
T classification				0.39
T1-T2	86 (61.4)	48 (64.9)	38 (57.6)	
T3-T4	54 (38.6)	26 (35.1)	28 (42.4)	
N classification				1.00
N0-N1	71 (50.7)	38 (51.4)	33 (50.0)	
N2-N3	69 (49.3)	36 (48.6)	33 (50.0)	
Chemotherapy				
Concurrent, yes	129 (92.1)	66 (89.2)	63 (95.5)	0.22
Adjuvant, yes	98 (70.0)	52 (70.3)	46 (69.7)	1.00
QoL completed				0.90
Before RT	140 (100.0)	74 (100.0)	66 (100.0)	
During RT (42.4 Gy)	137 (97.8)	72 (97.3)	65 (98.5)	
3 months after RT	130 (92.8)	69 (93.2)	61 (92.4)	
12 months after RT	104 (74.3)	55 (74.3)	49 (74.2)	

*RT* Radiotherapy, *QoL* Quality of life, *IMRT* Intensity modulated radiotherapy, *VMAT* Volumetric modulated arc therapy

### Mean dose of target volumes and OARs

Table 2 lists the mean dose (standard deviation) of the target volumes and OARs concerned. We observed no statistically significant differences in GTV, PTV-H, PTV-M, PTV-L and most of the maximal or mean dose of the OARs (except maximal dose of spinal cord) between the VMAT and IMRT group.

**Table 2:** Mean dose (standard deviation) of target volumes and normal organs at risk—VMAT versus IMRT

Variables	IMRT (*n* = 74)	VMAT (*n* = 66)	*P* value
GTV, mean	7458 (85)	7457 (84)	0.93
PTV-H, mean	7369 (49)	7363 (47)	0.44
PTV-M, mean	6826 (214)	6788 (172)	0.26
PTV-L, mean	5694 (178)	5660 (74)	0.25
Brain stem, maximum	5108 (419)	5094 (387)	0.84
Spinal cord, maximum	4188 (225)	4095 (215)	0.02
Right eyeball, mean	821 (489)	877 (269)	0.42
Left eyeball, mean	805 (289)	902 (319)	0.08
RT optic nerve	4244 (951)	4574 (978)	0.11
Lt optic nerve	4416 (1070)	4657 (809)	0.21
Right parotid, mean	2970 (568)	2912 (383)	0.51
Left parotid, mean	3017 (482)	2949 (463)	0.48
Oral cavity, mean	3796 (479)	3713 (428)	0.32
Larynx, mean	3976 (516)	3919 (431)	0.52
Cervical esophagus, mean	3712 (420)	3836 (455)	0.19

*IMRT* Intensity modulated radiotherapy, *VMAT* Volumetric modulated arc therapy, *GTV* Gross tumor volume, *PTV-H* High dose level of planning target volume, *PTV-M* Middle dose level of planning target volume, *PTV-L* Low dose level of planning target volume

### Locoregional control and survival

**Fig. 1:**
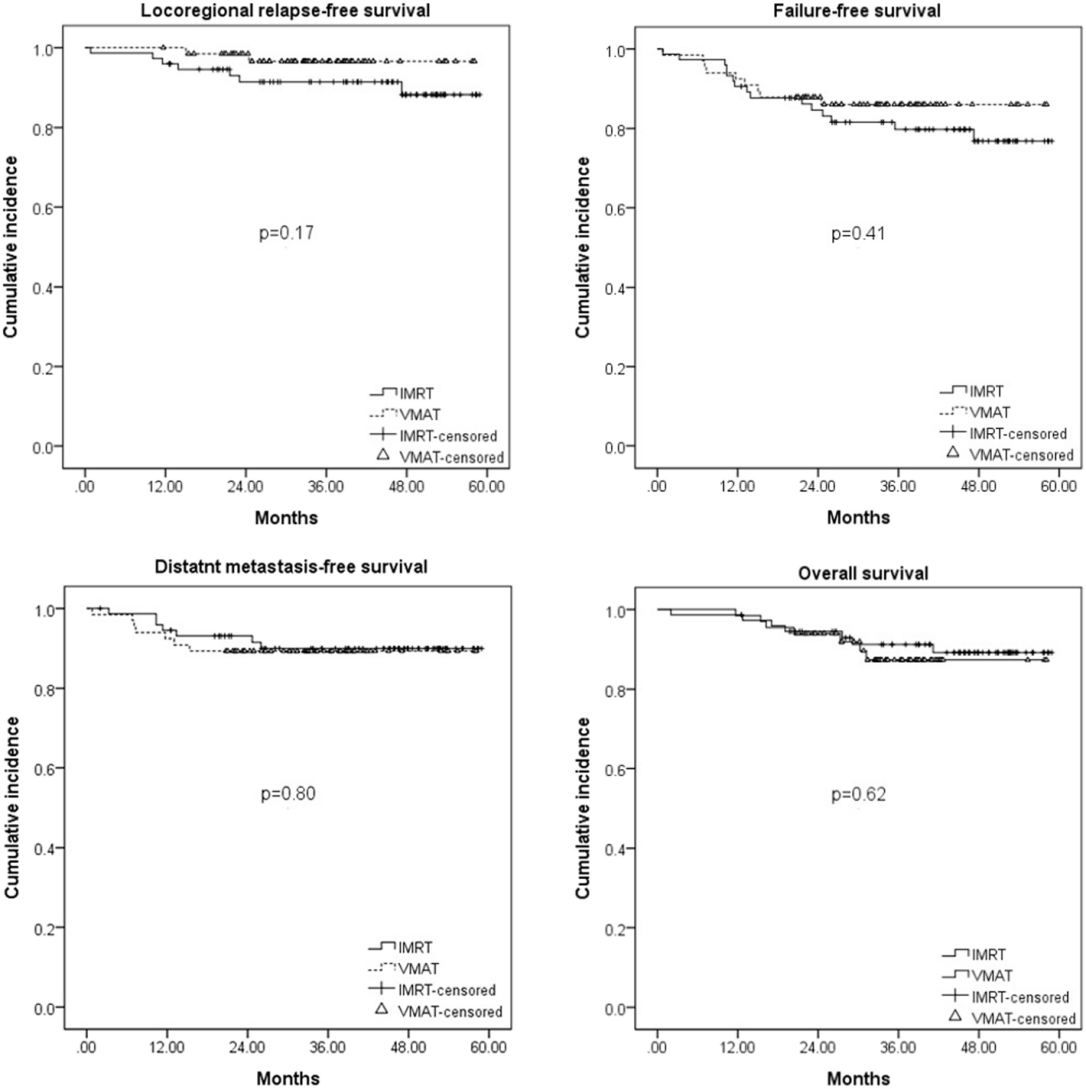
Survival comparisons of NPC patients treated by VMAT versus IMRT

The distributions of local failure, neck failure, and distant metastasis were one patient (1.5%), one patient (1.5%), and seven patients (10.6%) in the VMAT group, and six patients (8.1%), three patients (4.0%), and seven patients (9.5%) in the IMRT group, respectively. Ten patients died of the disease, three died of other medical diseases and one patient died of unknown causes. The 3-year LRRFS, DMFS, FFS, and OS were 96.6, 89.4, 86.1, and 87.4% for the VMAT group, respectively, compared with 91.4, 90.0, 79.8, and 91.3% for the IMRT group (*p* value > 0.05, Fig. 1). Further stratifying patients into subgroups by various categorical variables, we did not find any statistically significant difference in LRRFS, DMFS, FFS, or OS between the VMAT and IMRT groups. Entering the variables of socio-demographic variables, T&N classification, GTV, pre-treatment EBV DNA level (< 1500 vs ≧ 1500 copies/ml), RT technique, combination with adjuvant chemotherapy or not into multivariate analysis, we observed advanced N classification (N2–3 vs N0–1) was the only significantly negative prognosticator for all the four survival outcomes. The 3-year LRRFS, DMFS, FFS, and OS rates for those with N0–1 were 96.9, 94.0, 88.8, and 95.7% compared with 82.5, 82.2, 76.3, and 80.5% for those with N2–3 (all *p* values < 0.05), respectively. On the other hand, larger GTV was also observed to be predictive of poorer LRRFS. A 10 ml increase of GTV was associated with a 6% (95% CI, 1 to 11%, *p* = 0.014) increment in the likelihood of locoregional failure. A 10 years increase of age was associated with a 6% (95% CI, 1 to 11%, p = 0.014) increment in the likelihood of death.

### Changes of QoL for those who completed all questionnaires

**Fig. 2:**
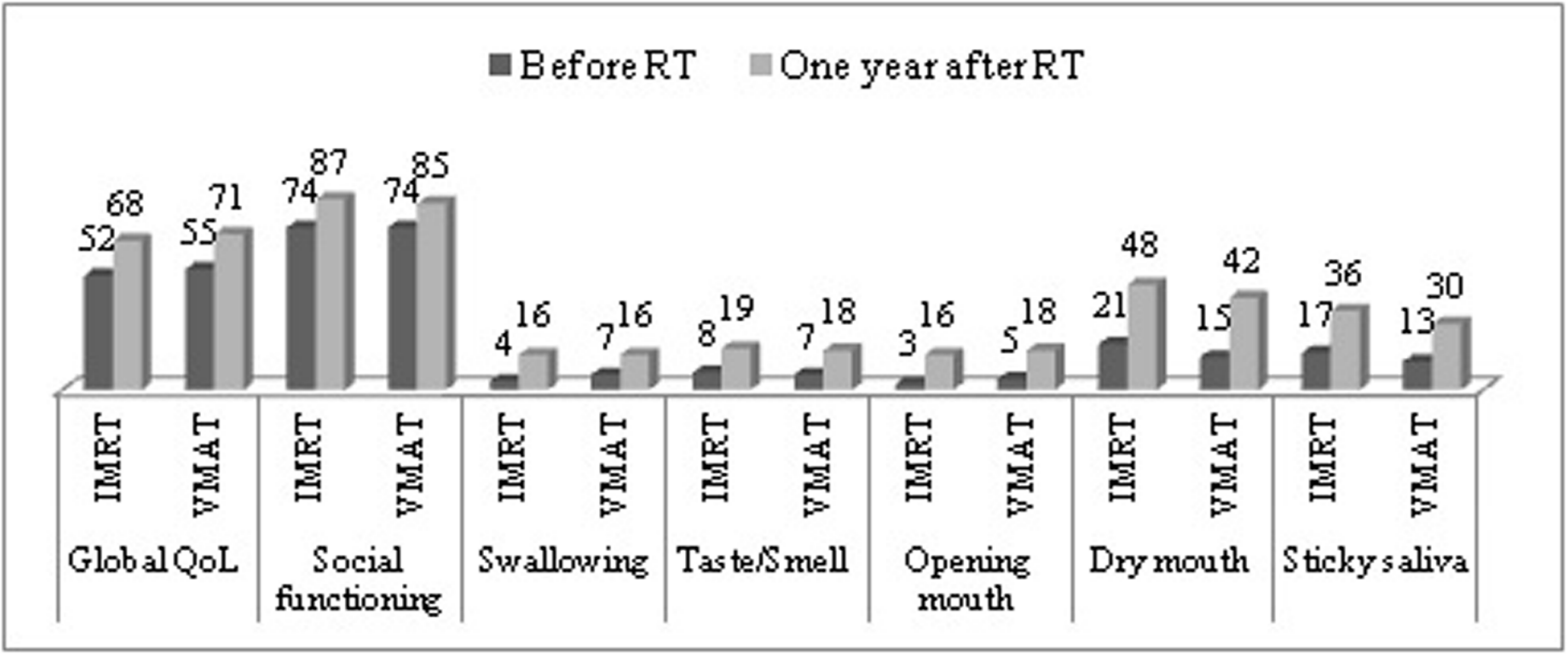
Mean scores of QoL scales with statistically (*p*<0.05) and clinically (difference≧10) significant difference between the time point of pre-RT and 1 year after RT for NPC patients treated by VMAT or IMRT

As regards the 104 patients who filled in all questionnaires at the four time points, as shown in Table 3, there were statistically significant changes of most scales of QLQ-C30 (except dyspnea, diarrhea, and financial difficulty) and HN35 (except teeth and coughing) over these periods. A general trend of maximal deterioration in most QoL scales was observed at ti2, followed by a gradual recovery thereafter. Global QoL, the five functional scales, some symptomatic problems of QLQ-C30, such as fatigue, nausea/vomiting, pain, insomnia, appetite loss, and constipation, and most symptomatic problems of HN35 recovered mainly between ti2 and ti3. Continuous recovery of global QoL, social functioning, fatigue, appetite loss, taste/smell, social eating, dry mouth, and sticky saliva was observed between ti3 and ti4. The pattern of QoL changes was similar between the VMAT and IMRT group. Compared with ti1, we observed statistically (*p*<0.05) and clinically (difference of mean scores≧10) better outcome in global QoL and social functioning, but worse head and neck symptomatic outcome in swallowing, taste/smell, opening mouth, dry mouth, and sticky saliva at ti4 (Fig. 2). Further analysis was performed to investigate any other medical- or socio-demographic- related parameters to exert a significant influence upon the changes of QoL scales following IMRT or VMAT, but no statistically significant predictor could be detected.

**Table 3:** Changes of quality of life for 104 NPC patients treated by IMRT or VMAT with completions of all questionnaires at the four time points

All	ti1	ti2	ti3	ti4	*p*-value
EORTC QLQ-C30					
Global quality of Life	53^†‡§^	34^¶※^	56^#^	67	< 0.01
Physical functioning	92^†‡^	81^¶※^	88	92	< 0.01
Role functioning	92^†‡^	70^¶※^	88	93	< 0.01
Emotional functioning	79^†‡^	72^¶※^	85	85	< 0.01
Cognitive functioning	85^†^	77^¶※^	85	85	< 0.01
Social functioning	75^†§^	66^¶※^	77^#^	87	< 0.01
Fatigue	21^†‡^	46^¶※^	29^#^	18	< 0.01
Nausea and vomiting	8^†^	42^¶※^	12	7	< 0.01
Pain	16^†^	39^¶※^	15	13	< 0.01
Dyspnea	8	11	8	6	NS
Insomnia	26	32^¶※^	21	22	< 0.01
Appetite loss	14^†‡^	62^¶※^	28^#^	13	< 0.01
Constipation	12^†^	27^¶※^	18	17	< 0.01
Diarrhea	11	12	10	8	NS
Financial difficulties	21	22	22	18	NS
EORTC QLQ-HN35					
Pain	5^†‡^	40^¶※^	12	7	< 0.01
Swallowing	5^†‡§^	42^¶※^	20	16	< 0.01
Senses (taste/smell)	8^†‡§^	51^¶※^	29^#^	17	< 0.01
Speech	6^†‡§^	27^¶※^	16	13	< 0.01
Social eating	4^†‡§^	48^¶※^	22^#^	11	< 0.01
Social contact	4^†‡^	20^¶※^	10	6	< 0.01
Sexuality	13^†‡§^	40^¶※^	25	22	< 0.01
Teeth	27^†^	29	26	27	NS
Opening mouth	4^†‡§^	22^¶※^	16	16	< 0.01
Dry mouth	20^†‡§^	62^¶※^	54^#^	44	< 0.01
Sticky saliva	16^†‡§^	61^¶※^	45^#^	34	< 0.01
Coughing	18^†^	22	18	19	NS
Feeling ill	24^†^	55^¶※^	27	22	< 0.01

Analysis by multivariate ANOVA for repeated measures; *IMRT* Intensity modulated radiotherapy, *VMAT* Volumetric modulated arc therapy; ti1: before RT; ti2: RT 42.4 Gy; ti3: 3 months after RT; ti4: 12 months after RT; †: *p* < 0.05, ti1 vs. ti2; ‡: *p* < 0.05, ti1 vs. ti3; §: *p* < 0.05, ti1 vs. ti4; ¶: *p* < 0.05, ti2 vs. ti3; ※: *p* < 0.05, ti2 vs. ti4; #: *p* < 0.05, ti3 vs. ti4; NS: not statistically significant

### QoL at each time point

The comparisons of the mean scores of each QoL scale for patients who completed the QoL questionnaire at each time point between the two groups were demonstrated in Table 4. We observed there was no statistically or clinically significant difference in all the QoL scales at each time point for those patients treated by VMAT versus IMRT. Further stratifying patients into subgroups by various categorical variables, we still did not find any statistically or clinically significant difference in the QoL outcome at each time point between the IMRT and VMAT.

**Table 4:** Quality of life at each time points: IMRT vs. VMAT

	IMRT: VMAT			
	ti1	ti2	ti3	ti4
Patient number	74: 66	72: 65	69: 61	55: 49
EORTC QLQ-C30				
Global quality of life	54: 55	34: 40	57: 60	67: 66
Physical functioning	91: 94	81: 80	86: 87	92: 90
Role functioning	77: 75	68: 65	78: 76	86: 87
Emotional functioning	91: 93	73: 72	89: 87	96: 92
Cognitive functioning	79: 73	75: 74	85: 82	86: 87
Social functioning	84: 87	77: 80	84: 85	87: 83
Fatigue	19: 22	42: 48	29: 29	20: 25
Nausea/Vomiting	7: 7	42: 40	10: 9	5: 2
Pain	13: 16	38: 35	17: 14	13: 15
Dyspnea	9: 4	13: 13	11: 6	7: 3
Insomnia	21: 25	28: 34	19: 25	19: 24
Appetite loss	13: 13	59: 59	27: 22	12: 9
Constipation	13: 11	25: 23	16: 19	13: 15
Diarrhea	8: 12	14: 15	9: 9	8: 5
Financial difficulties	25: 24	28: 25	22: 25	17: 12
EORTC QLQ-HN35				
Pain	7: 5	38: 35	15: 15	10: 7
Swallowing	7: 5	44: 40	20: 18	14: 15
Senses (taste/smell)	7: 6	46: 50	27: 24	16: 14
Speech	7: 4	20: 18	17: 14	11: 7
Social eating	5: 2	46: 42	20: 20	10: 13
Social contact	4: 3	20: 17	11: 10	5: 5
Sexuality	14: 17	39: 42	23: 27	16: 22
Teeth	18: 22	28: 31	23: 27	25: 28
Opening mouth	5: 3	18: 14	14: 13	12: 15
Dry mouth	18: 2 1	59: 57	53: 51	44: 46
Sticky saliva	15: 16	55: 58	39: 3 9	30: 27
Coughing	20: 18	21: 24	22: 21	18: 23
Feeling ill	23: 16	50: 54	27: 28	17: 18

*IMRT* Intensity modulated radiotherapy, *VMAT* Volumetric modulated arc therapy; ti1 = before RT; ti2: RT 42.4 Gy; ti3: 3 months after RT; ti4: 12 months after RT.

## Discussion

IMRT has been the standard treatment in NPC patients for many years. In the past decades, more advanced RT technique especially the rotational arc technique either by linear accelerator or helical tomotherapy has been made with an attempt to further increment the therapeutic window of RT in treating NPC. Because previous reports suggest the noteworthy incremental improvement in dose distributions of VMAT over IMRT, the comparison of treatment outcome for patients treated by the two techniques becomes encouraging. To our knowledge, this is the first study to compare the clinical outcome for patients with NPC treated by VMAT versus IMRT.

Although nonrandomized but with comparable components of patients, disease and treatment characteristics between both groups in current study, we fail to see a statistically significant improvement in the 3-year LRRFS, DMFS, FFS and OS comparing VMAT with IMRT. Our survival outcome was comparable to the series of VMAT published by Guo et al. [15] or other series of IMRT studies [6, 9, 16]. With identical mean dose at GTV and the three dose levels of PTV between the two groups, it is not surprising that no significant difference in tumor control or patient survival was observed.

Meanwhile, with similar mean or maximal dose of most OARs concerned, we observed no significant difference between the two groups in all the QoL scales at the follow-up time points. The most noteworthy advantage of IMRT or VMAT in the treatment of NPC appears to be related to its ability to preserve salivary function. Salivary dysfunction has been observed to be correlated to the mean dose of parotid gland, and long-term salivary dysfunction is usually preventable if the mean dose of one parotid gland could be spared to less than 20 Gy or both glands spared to less than 25 Gy [17]. With parotid sparing IMRT, many clinical studies have proved the therapeutic benefit of IMRT in preserving salivary function relative to the conventional technique [16, 18, 19]. Whether further reduction of the mean dose in parotids could be achieved by using VMAT in NPC is contradictory in different studies. In our patients, the mean dose of both parotids is around 30 Gy by either VMAT or IMRT. In the study by Lu et al. [1], they compared the dosimetric outcome between the planning of VMAT and IMRT using the same NPC patients and observed a statistically significant reduction (from 31.3Gy to 26.3 Gy) of the mean dose in parotids, however, the result could not be repeated in similar studies reported by Jin et al. [20] or Lee et al. [7].

It is difficult to interpret how much dose reduction of OARs is needed to transfer into the clinical benefits as revealed in the QoL scales in the literature. In a randomized trial to compare IMRT with 2D-RT for early stage NPC, with a remarkable dose reduction of parotids from 61.5 Gy to 32.2 Gy, IMRT results in significantly less observer-rated delayed xerostomia, but not patient-reported xerostomia [19]. In our previous study to compare IMRT with 3D-CRT in NPC patients, with a moderate dose reduction of 12–13 Gy in parotids and oral cavity and 5–8 Gy at the OARs of skull base in IMRT group, a significant improvement in global QoL, fatigue, taste/smell, dry mouth, and feeling ill was observed at the time point of 3 months after RT but the improvement disappeared at the longer follow-up time points [21]. On the contrast, with a mild dose reduction of parotids from 34.1 Gy to 27.3 Gy by helical tomotheapy compared to IMRT, Chen et al. observed a statistically significant difference of 7% versus 38% in their NPC patients who subjectively reported “too little” or “no” saliva at final follow-up [22].

We observed a maximal deterioration of most QoL scales during treatment by IMRT or VMAT, followed by a gradual recovery thereafter. Such a pattern of QoL changes was also observed in other HNC patients following IMRT or 3D-CRT [23]. It has been reported that rehabilitation after multimodal treatment for HNC takes 1 year or more and some QoL scales might have improved considerably, but others, especially in some HNC-specific domains remain compromised [24]. Tribius et al. observed most QoL scales would return to the baseline level but some residual deficits, e.g. dry mouth and sticky saliva were still persistent 1 year later for locally advanced HNC patients treated by definite IMRT [25]. For NPC patients treated by IMRT or VMAT as revealed in the study, we observed global QoL and social functioning recovered to significantly better than the pre-treatment status, but the symptomatic scales in swallowing, taste/smell, opening mouth, dry mouth, and sticky saliva were still worse. Changes in QoL following treatment might reflect the combined effects of tumor regression and treatment related complications that patients have perceived. Before treatment, patients had just received the catastrophic information of being diagnosed with cancer and were facing a possibly life-threatening treatment, which might have great impact on their global health status. After having finished the treatment, experienced tumor shrinkage, and recovered from acute side effects, they had time to adapt to the situation, though some late complications in head-and-neck area still remained. Furthermore, the adaptation process called response shift might have occurred during the recovery process, and patients might have reappraised their life domains and altered satisfaction with life themes [21].

With lack of randomization, this study has several limitations. We cannot rule out the presence of some unmeasured confounding factors (e.g. the positional or volumetric changes during the treatment course) between the two groups and the inevitable bias imposed by different operators of the treatment planning systems. Except for the mean dose, detailed dosimetric data were not provided in the cohort; therefore, it was difficult to establish the specific dose-response relationship at the target and OARs that might have contributed to the clinical outcomes observed between the two techniques. In the study, the orthogonal KV X-ray images were used to perform the daily image guidance to overcome organ motion and daily treatment set-up uncertainties, which could potentially be improved by daily cone beam CT or MR guidance [26, 27].

In conclusion, the study provides the evidence that the tumor control, survival and changes of QoL is compatible for NPC patients treated by VMAT versus IMRT.

## Data Availability

The datasets used and/or analyzed during the current study are available from the corresponding author on reasonable request.

## Abbreviations

QoL: Quality of life
NPC: Nasopharyngeal carcinoma
VMAT: Volumetric-modulated arc therapy
IMRT: Intensity-modulated radiotherapy
OARs: Organs at risk
EORTC QLQ-C30 and HN35 module: European Organisation for Research and Treatment of Cancer Quality of Life Questionnaire-Core 30 Head and Neck 35-questions
LRRFS: Locoregional relapse-free survival
DMFS: Distant metastasis-free survival
FFS: Failure-free survival
OS: Overall survival
GTV: Gross tumor volume
CTV: Clinical target volume
PTV: Planning target volume
RTOG: Radiation Therapy Oncology Group
MLCs: multi-leaf collimators
EBV: Epstein-Barr virus
